# Distinguishing between limited systemic scleroderma-associated pseudo-obstruction and peritoneal dissemination

**DOI:** 10.1186/s40792-014-0010-4

**Published:** 2015-02-24

**Authors:** Susumu Saigusa, Yasuhiro Inoue, Masaki Ohi, Hiroki Imaoka, Ryo Uratani, Minako Kobayashi, Masato Kusunoki

**Affiliations:** Department of Surgery, Wakaba Hospital, 28-13 Minami-Chuo, Tsu, Mie 514-0832 Japan; Department of Gastrointestinal and Pediatric Surgery, Mie University Graduate School of Medicine, Tsu, Mie Japan

**Keywords:** Pseudo-obstruction, Peritoneal recurrence, Limited systemic scleroderma, FDG PET-CT

## Abstract

A 78-year-old woman receiving treatment for limited systemic scleroderma (SSc) underwent high anterior resection and partial liver resections for rectosigmoid colon cancer with multiple liver metastases. A year after surgery, an abdominal computed tomography (CT) demonstrated suspicion for peritoneal dissemination with an increase in ascites, and ^18^F-fluorodeoxy glucose-positron emission tomography-CT was suggestive of carcinomatosis. We began to decompress the small intestine and administer octreotide. However, the intestinal obstruction did not improve. Although intestinal pseudo-obstruction caused by limited SSc was considered as a differential diagnosis, we performed an exploratory laparotomy because the possibility of peritoneal dissemination-associated obstruction could not be excluded. We observed a moderate amount of serous ascites and dilatation of the small intestine that was white in color, hard, and with limited contractility. There was no evidence of peritoneal dissemination nor of mechanical obstruction. Our experience thus shows the difficulty of distinguishing SSc-associated intestinal pseudo-obstruction from peritoneal dissemination.

## Background

Intestinal pseudo-obstruction is caused by several diseases including connective tissue disorders, hypothyroidism, Chagas’ disease, diabetes, and Parkinson’s disease. Systemic scleroderma (SSc) is a chronic disorder of the connective tissue characterized by inflammation, fibrosis, and degeneration of the skin and blood vessels and is known to cause intestinal pseudo-obstruction [1-5]. The radiographic findings of intestinal pseudo-obstruction caused by SSc are a hide-bound bowel sign or accordion sign on abdominal X-ray or contrast study and pneumatosis cystoides intestinalis on abdominal computed tomography (CT) [3,6-8]. However, these findings cannot exclude mechanical obstruction such as that caused by postoperative adhesions or peritoneal dissemination. Although ^18^F-fluorodeoxy glucose-positron emission tomography (FDG PET-CT) is a useful tool to detect peritoneal metastases, low-grade FDG uptake should be carefully assessed because it is difficult to differentiate inflammation or fibrotic change from tumor recurrence [9-12]. We report a case in which it was difficult to distinguish between SSc-associated pseudo-obstruction and peritoneal dissemination in a SSc patient after surgery for stage IV colon cancer.

## Case presentation

**Figure 1:**
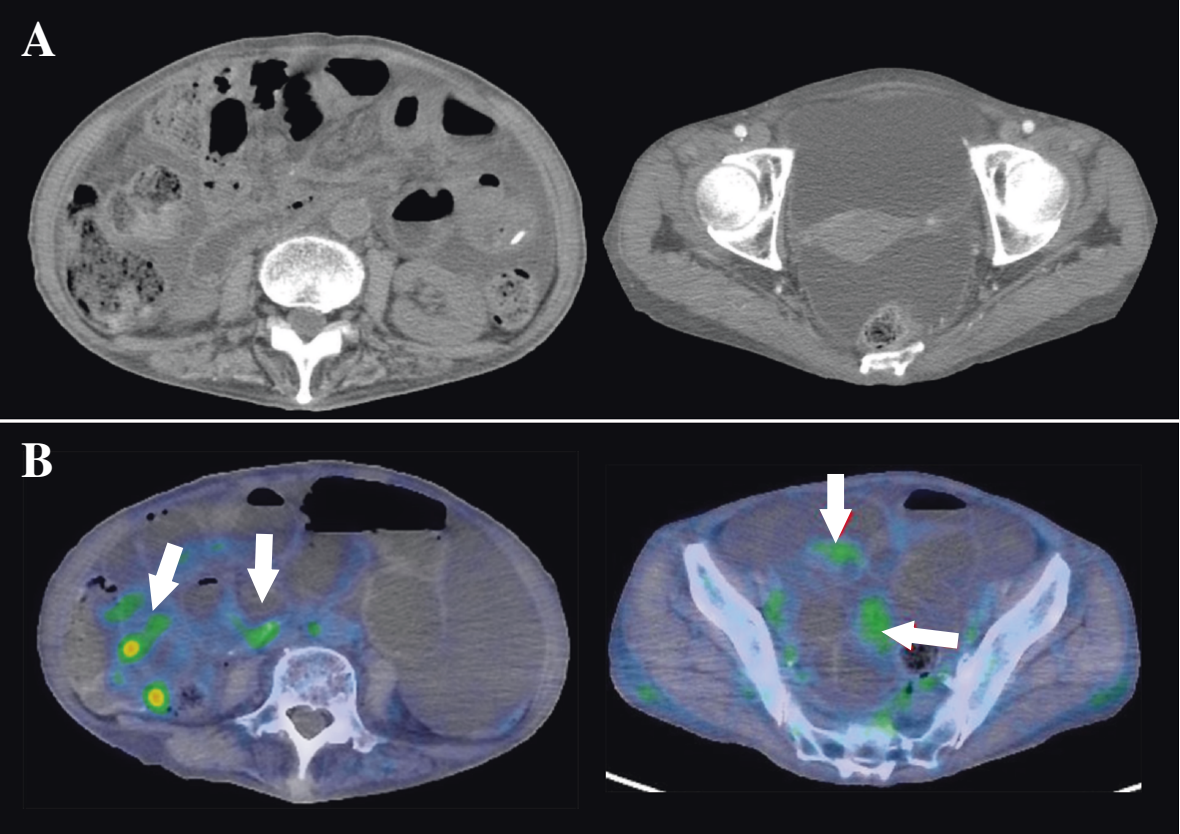
**Increase in ascites and diffuse low-grade FDG uptake.** Abdominal CT shows an increase in ascites and mild thickness of the small intestinal wall **(A)**. Diffuse low-grade FDG uptake along the small intestinal wall (white arrows) **(B)**.

**Figure 2:**
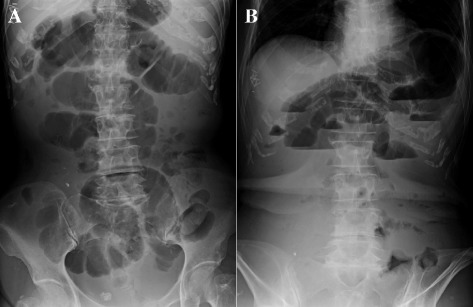
**Abdominal X-ray before surgery.** Upright position **(A)**, supine position **(B)**.

**Figure 3:**
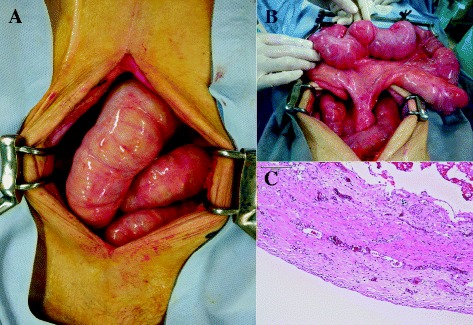
**Operative and histopathological findings.** Dilatation and hardening of the small intestine **(A)**. Diffuse fibrotic change throughout the small intestine **(B)**. Fibrotic change in the muscle and serosal layers **(C)**. Hematoxylin and eosin stain, original magnification × 40.

A 78-year-old woman receiving treatments including the administration of low dose of corticosteroid and proton pump inhibitor for LSSc (antinuclear antibody: positive; anti-centromere antibody: positive; anti-topoisomerase antibody: negative; anti-ribonucleoprotein antibody: negative) for 7 years underwent high anterior resection and partial liver resections for rectosigmoid colon cancer with multiple liver metastases (S4, S6, and S8). The preoperative serum levels of carcinoembryonic antigen (CEA) and carbohydrate antigen (CA19-9) were 7.9 ng/ml (normal range, ≤5.8 ng/ml) and 85.7 U/ml (normal range, 0 to 37.0 U/ml). Pathological findings showed well-differentiated tubular adenocarcinoma, T3N1M1 with lymphatic invasion. On partial resection for each liver metastasis, S4 lesion resulted in positive resection margin. Although she received adjuvant therapy (capecitabine plus oxaliplatin) for 6 months after surgery, she stopped the therapy because of the side effects. A year after the surgery, an abdominal CT demonstrated suspected peritoneal dissemination with increased ascites (Figure 1A). Tumor markers were not increased (both CEA and CA19-9 were within normal range), but the ascites was increasing and obstructive symptoms such as nausea, emesis, and abdominal distension worsened. Contrast study showed delayed transit through the small intestine with eventual movement into the colon. To confirm whether or not there was any evidence of tumor recurrence, we performed FDG PET-CT, which suggested peritonitis carcinomatosa with diffuse low-grade FDG uptake along the small intestinal wall (Figure 1B). We began to decompress the small intestine and administer octreotide. However, the intestinal dilatation and stasis did not improve (Figure 2). Although pseudo-obstruction caused by LSSc was considered as a differential diagnosis, we performed an exploratory laparotomy because the possibility of partial obstruction caused by peritoneal dissemination could not be excluded and the patient failed to improve with conservative measures. Preoperative laboratory investigation revealed anemia (hemoglobin 9.6 g/dl), mild elevated liver enzymes (aspirate aminotransferase 43 IU/l, alanine aminotransferase 47 IU/l), and poor nutritional condition (total protein 4.9 g/dl, albumin 2.9 g/dl).Upon laparotomy, we observed a moderate amount of serous ascites and dilatation of the small intestine overlying which was a whitish film. The bowel was hardened, with wall thickness and limited contractility, similar to findings in encapsulating peritoneal sclerosis (Figure 3A,B). Its findings were observed in all the small intestine. However, there was no evidence of peritoneal dissemination or mechanical obstruction. Eventually, we performed an enterostomy and sampling of the small intestinal serosa. On histopathological examination, fibrotic change in the muscle and serosal layers was observed (Figure 3C). Postoperatively, we administrated neostigmine bromide as a substitute for octreotide (not approved for this indication in Japan) and erythromycin to promote intestinal motility, but these treatments were not effective. We treated her with the decompression of the small intestine via enterostomy for stasis and total parenteral nutrition for stasis-related malabsorption and malnutrition. However, her general condition such as anasarca induced by malnutrition was gradually worse. Ultimately, she suffered from sepsis caused by stasis-related bacterial overgrowth and translocation, and died on postoperative day 77.

## Conclusions

Gastrointestinal involvement is the most common non-dermatologic complication in patients with SSc [3,13]. Gastrointestinal dysfunction can severely and negatively impact quality of life and is associated with a poor prognosis [14-18]. Limited SSc is a subtype of systemic scleroderma that was previously referred to as CREST syndrome (calcinosis, Raynaud’s phenomenon, esophageal dysmotility, sclerodactyly, telangiectasias). Patients with limited SSc develop sclerosis of the skin distal to their elbows, knees, and face and are more likely to develop pulmonary hypertension. Patients with another subtype of SSc, diffuse SSc, develop not only distal but also proximal sclerosis and tend to have more significant visceral organ involvement than those with limited SSc. However, gastrointestinal involvement is common in both subtypes and the severity of cutaneous and gastrointestinal manifestations often does not correlate [3,19,20]. Pathologic manifestations in patients with SSc are due to progressive fibrosis of intestinal smooth muscle. Physiologically, inflammation of the myenteric ganglia and fibrosis of gastrointestinal muscle have been found in patients with SSc [21,22]. Small intestinal hypomotility causes luminal dilatation and stasis of luminal contents resulting in a pseudo-obstruction, leading to stasis-related malabsorption. Secondary small intestinal bacterial outgrowth affects almost half of patients with SSc [5,23-25]. It has been reported that octreotide is the most potent prokinetic drug for pseudo-obstruction [26,27]. However, octreotide did not improve motility in the present case. Moreover, erythromycin, which mimics motilin, known as the prokinetic hormone, was also not effective, despite the fact that several authors have reported upon its effectiveness for pseudo-obstruction [28,29]. We think that the lack of effectiveness of these drugs is due to the progressive fibrosis of the small intestine and malabsorption by small intestinal bacterial overgrowth. Although we tried enteral nutrition in the form of an elemental diet via the enterostomy with concomitant administration of antibiotics such as metronidazole [30,31], the nutrition status of our patient did not improve. We think that early diagnosis and treatment for pseudo-obstruction is important, along with the collaboration of rheumatologists and gastroenterologists in patients with gastrointestinal involvement of SSc. Although we lack a complete understanding of the progressive nature of the disease, the presence of carcinoma in addition to adjuvant chemotherapy may compound the situation [32].

On radiographic examination, abdominal X-ray did not show the typical appearance of SSc-associated pseudo-obstruction such as the hide-bound bowel sign [3,6-8]. Although we observed pneumatosis cystoides intestinalis in the descending colon 2 months before exploratory laparotomy, this finding was not noted during the surgery. Given the increase in ascites and occurrence of obstructive symptoms, most would suspect peritoneal recurrence in a patient with stage IV colon cancer after surgery. FDG PET-CT has the potential to improve detection of peritoneal carcinomatosa [9-12]. However, FDG uptake is also present in cases of fibrosis and inflammation, and it is difficult to distinguish this pathology from peritoneal metastasis [9-12]. Although diffuse low-grade FDG uptake (up to the maximum standardized uptake value of 2.3) along the small intestinal wall was observed, we were unable to definitively diagnose the current case as one of intestinal pseudo-obstruction. Several authors reported that evaluation for radiation-induced fibrosis and retroperitoneal fibrosis is helpful [33-35]. On the other hand, Nishiyama et al. have reported that FDG PET-CT is useful to evaluate the degree of disease associated with connective tissue disorder [36]. In the current case, we think that elevated FDG uptake along the small intestinal wall was indicative of inflammation and fibrosis.

In conclusion, our experience suggests that it is difficult to distinguish LSSc-associated intestinal pseudo-obstruction from peritoneal dissemination. Although FDG PET-CT is an effective tool for detection of peritoneal metastasis, comprehensive assessment of the findings in a patient with both SSc and advanced gastrointestinal cancer is necessary.

## Consent

Written informed consent was obtained from the patient’s husband for publication of this case report and any accompanying images. A copy of the written consent is available for review by the Editor-in-Chief of this journal.
